# COVID-19 Bereavement: Considerations for Families, Psychologists, and Student Trainees

**DOI:** 10.1093/geroni/igaa057.3436

**Published:** 2020-12-16

**Authors:** Jacqueline Hogan, Lisa Richards

**Affiliations:** 1 University of Massachusetts Boston, Boston, Massachusetts, United States; 2 Bedford VA Medical Center, Bedford, Massachusetts, United States

## Abstract

During the peak of COVID-19, inflicted individuals died unexpectedly and in isolation. These circumstances deprived families of opportunities to say goodbye or memorialize the death of their loved one in alignment with their values and cultural heritage (e.g., wakes/vigils, funerals, shiva, washing, shrouding, military honors). Unable to hold hands, have final conversations, or develop treatment plans with providers, bereaved families experienced compounded losses. Concurrent quarantine hindered their engagement in coping strategies. COVID-19 bereavement increases the risk for complicated grief, which escalates the risk of physical and mental health problems, suicide, drug abuse, and family discord (Shear, 2015, 2020). While death, grief, and mourning are normal life experiences, traumatic and sudden death during a global pandemic is a new domain and the voices of those left behind are under-represented in social discourse. Simultaneously, psychologists and trainees quickly became last responders. COVID-19 presented a constellation of clinical challenges. Practitioners provided care during a time of political and racial tension, civil unrest, school closures, health and financial insecurity, and a collective loss of normalcy. Additionally, COVID-19 cast a spotlight on ageist attitudes and critical need for increased representation of older adults in training curricula. These issues echo the call to embrace aging as a valued aspect of diversity, and to strengthen psychology’s workforce in the areas of training, practice, research and advocacy for aging adults (Hoge, et al., 2015). This poster will explore the impact of COVID-19 bereavement on families and practitioners, promote advocacy efforts, and offer tangible training recommendations for psychology programs.

